# Myocardial Postsystolic Shortening and Early Systolic Lengthening: Current Status and Future Directions

**DOI:** 10.3390/diagnostics11081428

**Published:** 2021-08-06

**Authors:** Philip Brainin

**Affiliations:** Department of Cardiology, Herlev-Gentofte Hospital, Copenhagen University Hospital, DK-2900 Gentofte, Denmark; pjen0286@regionh.dk

**Keywords:** deformation, postsystolic shortening, early systolic lengthening, speckle tracking echocardiography, clinical value

## Abstract

The concept of paradoxical myocardial deformation, commonly referred to as postsystolic shortening and early systolic lengthening, was originally described in the 1970s when assessed by invasive cardiac methods, such as ventriculograms, in patients with ischemia and animal experimental models. Today, novel tissue-based imaging technology has revealed that these phenomena occur far more frequently than first described. This article defines these deformational patterns, summarizes current knowledge about their existence and highlights the clinical potential associated with their understanding.

## 1. Introduction

In the past two decades, non-invasive imaging for assessment of cardiac function has become increasingly widespread in cardiology. In particular, the use of echocardiography, a key examination, has undergone rapid technological developments [1,2]. Traditionally, global and regional left ventricular function is often based on visual inspection of the cardiac wall segments. However, this is qualitative and subjective and often requires significant clinical experience to minimize intra- and inter-observer bias [3,4].

More recent tissue-based imaging technology has provided a more objective measure of cardiac function [5] and allows identification of specific deformational patterns, including postsystolic shortening (PSS) and early systolic lengthening (ESL) [6]. Today, PSS has been described in a plethora of studies, whereas ESL is more sparsely investigated. Both patterns represent a paradoxical deformation of the myocardial wall segments, and their identification may be useful for early recognition of ischemia, ischemic memory imaging and evaluating prognosis [6,7]. Considering the continuous interest in these patterns, an appraisal of the most recent literature on this topic is timely. The aim is to provide an overview of the contemporary knowledge on PSS and ESL and the potential clinical applicability of these patterns—spanning from normal myocardium to acute and chronic ischemia. Future directions and issues that need to be resolved to implement these features in a clinical setting will be highlighted. 

## 2. Physiological Deformation

Is paradoxical deformation a feature of normal cardiac physiology? During ejection phase, it is estimated that approximately one-third of all wall segments in healthy individuals display PSS [8], defined as a delayed contraction after aortic valve closure (also known as postsystolic thickening when assessed by radial strain). In general, physiological PSS has a small magnitude and short duration [8,9]. By nature, timing of end-systole is crucial to accurately delineate PSS from systolic shortening. The same applies to ESL, defined as a lengthening in early systole (also referred to as systolic bulging), for which timing of end-diastole is important. Even though both deformation patterns do not contribute to the ejection of blood, their existence have been related to physiological reshaping of the left ventricle in healthy individuals [8,10]. A growing body of literature suggests that both patterns in a normal myocardium are influenced by age, sex and loading conditions [11]. Additionally, both deformation patterns are prone to changes in loading conditions, such that they are amplified with higher afterload and reduced with greater preload [12,13]. 

**Figure 1:**
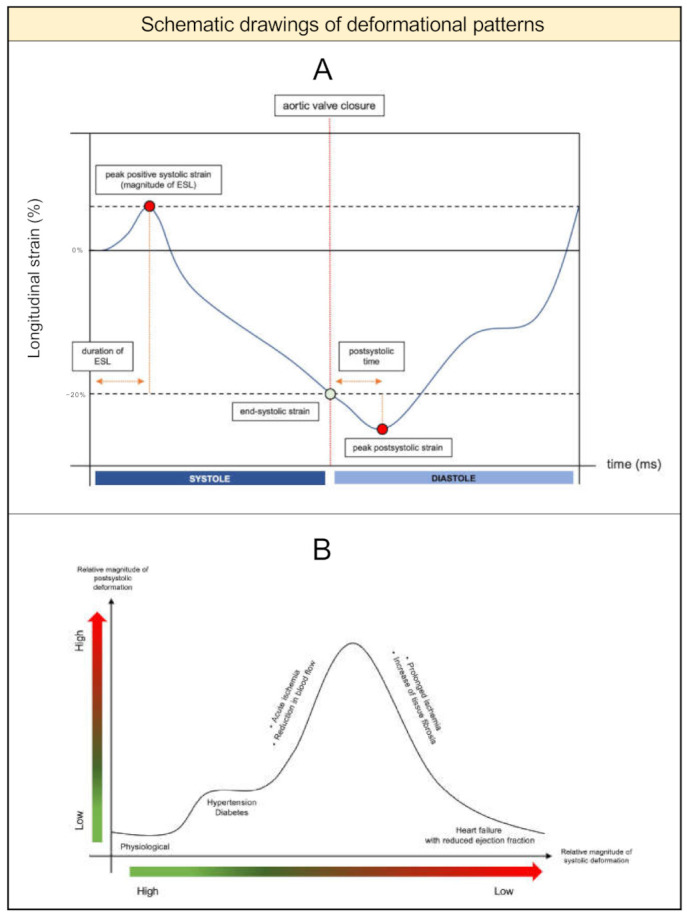
Longitudinal speckle tracking profile and suggested natural history of postsystolic shortening. Legend: (**A**): Schematic drawing of longitudinal speckle tracking profile displaying early systolic lengthening and postsystolic shortening as indicated with red dots. (**B**): Suggested natural history of postsystolic shortening. Relative magnitude of postsystolic shortening throughout different populations as a function of relative magnitude of systolic deformation. ESL: early systolic lengthening.

**Figure 2:**
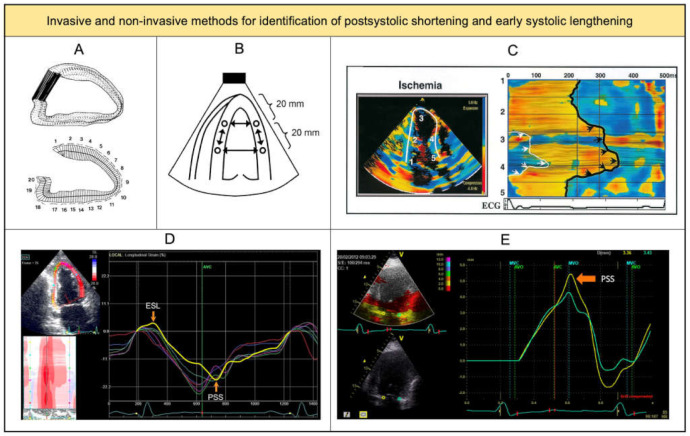
Invasive and non-invasive methods for assessing deformation patterns. Legend: (**A**) Ventriculogram depicting the centerline method for assessing wall motion. (**B**) Circles in the myocardial walls indicate embedded ultrasonic crystals to determine segmental length during the cardiac cycle. (**C**) Strain rate map during ischemia, where black arrows indicate postsystolic shortening and white arrows indicate delayed systolic shortening. (**D**) Longitudinal speckle tracking profile showing early systolic lengthening and postsystolic shortening. (**E**) Tissue Doppler imaging of longitudinal displacement of the mitral valve annulus showing postsystolic shortening. (**A**–**C**) are reprinted from Hosokawa et al. [14], Amundsen et al. [15] and Pislaru et al. [16] with permission the American College of Cardiology (Elsevier). (**D**) is reprinted from Asanuma et al. [6] with permission from the British Medical Journal Publishing Group. (**E**) is reprinted from Brainin et al. [17] with permission from Springer Nature.

In clinical research, the following indices are widely used for quantification: postsystolic index ([peak postsystolic strain]—[end-systolic strain])/[peak postsystolic strain] and early systolic index: [peak positive systolic strain]/[end-systolic strain] (Figure 1A). Postsystolic time, defined as the interval from aortic valve closure to peak postsystolic strain, and duration of ESL, defined as time from Q-wave on the ECG to peak positive systolic strain, are also used. Throughout the last decades, several imaging methods have been used to quantify both patterns, involving invasive methods (ventriculograms, sonomicrometry) [14,15] and non-invasive methods (strain-rate, tissue Doppler imaging, speckle tracking) (Figure 2A–E).

### 2.1. Conditions That Affect Deformation

Multiple parameters affect myocardial deformation [18,19]. Intrinsic contractility determines the contractile force and deformation of each myocardial segment and is influenced by electrical activation [20] and tissue perfusion. Consequently, deformation patterns in the setting of bundle branch block and in pace-rhythms should be interpreted with caution. As myocardial segments are embedded in the walls of the ventricle, surrounding segments may employ external forces on each other and alter the deformation. External forces are caused by cavity pressure (preload/afterload), which determines wall stress and tension from neighboring segments [9]. This is mostly an issued related to tissue Doppler imaging, as speckle tracking theoretically is less affected by myocardial tethering. Elasticity, an essential component in tissue structure, is affected by fibrosis and relates directly to deformation. This was demonstrated in a study where PSS was associated with procollagen type 1 carboxyterminal propeptide, a marker of myocardial fibrosis [21]. When tissue elasticity is replaced by fibrosis, it leads to a gradual decline in deformation, up to the point where segments only move passively. Because deformation implies that elasticity to some degree is preserved, PSS and ESL have been coined as predictors of tissue viability [22,23,24,25,26]. Other parameters that affect deformation patterns are pharmacotherapy (dobutamine, beta-blockers) [27] and possibly also diabetes and hypertension [21,28].

### 2.2. Proposed Underlying Mechanisms

Although no consensus exists, there has been considerable interest in the mechanisms accounting for PSS. The most controversial topic is whether PSS represents active contraction or passive recoil. By assessing left ventricular pressure and segmental length loops before and after coronary stenosis and occlusion, Skulstad et al. concluded that dyskinetic segments display PSS by a passive mechanism, whereas akinetic and hypokinetic segments rely on active contraction [29]. Bijnens et al. argued that this method takes into account external forces acting upon the ischemic segments [27]. Thus, segmental length loops cannot be strictly used to answer this question. In support of active contraction, Lyseggen and colleagues implemented a lengthening–shortening ratio with the purpose of distinguishing active and viable segments from passive segments, albeit it was recognized that passive segments still can be viable [30,31]. By contrast, Claus et al. established a mathematical model and found that PSS during ischemia largely can be explained by passive recoil [32], a finding also supported by Akaishi et al. [33]. No studies have specifically addressed the underlying mechanisms of ESL.

### 2.3. Association to Filling Pressure

PSS may lead to abnormal intraventricular filling [12], subsequently delaying diastolic lengthening and contributing to increased left ventricular filling pressure. Studies have demonstrated how PSS correlates with echocardiographic indices of diastolic function such as E/A-ratio, E/e’-ratio and diastolic strain rate [11,21,34]. Likewise, PSS appears to be more pronounced in hypertensive patients compared with healthy individuals, which could be due to a greater risk of subclinical ischemic heart disease among hypertensives [21,34,35]. Whether PSS contributes to development of diastolic dysfunction remains uninvestigated.

### 2.4. Phenotype of Paradoxical Deformation

Presence of physiological PSS and ESL can complicate timely identification of pathological PSS and ESL. Similarly, it is difficult to assess PSS and ESL in already damaged and fibrotic myocardial tissue. Criteria to delineate physiological versus pathological PSS were proposed by Voigt et al. (Table 1) [8], and it was suggested that evaluating strain rate during the isovolumic relaxation time could be used for discrimination [9,36]. Regardless of this, the relative amount of PSS seems to increase across populations with a greater burden of cardiovascular diseases [11,17,37,38]. Similar approaches for differentiating ESL are encouraged in future clinical investigations.

While pathological PSS and ESL commonly are seen as features of ischemic myocardium, it is noteworthy that they are also observed in non-ischemic heart disease, such as hypertrophic and dilated cardiomyopathy [39,40], Takotsubo cardiomyopathy [41], hypertension [21,34,35], aortic stenosis [42] and in electrical conduction delay [20,43,44]. Ring et al. showed that the presence of PSS correlates with the duration of the QRS complex [45]. In fact, it is regarded that these deformational patterns may be induced by any condition yielding an imbalance, or heterogeneity, between neighboring myocardial segments, irrespective of whether this is caused by ischemia, higher wall stress or delayed contraction. Thus, PSS and ESL are sensitive for detecting ischemia but offer little specificity.

## 3. Pathological Deformation

### 3.1. Evaluation in Acute Ischemia

**Figure 3:**
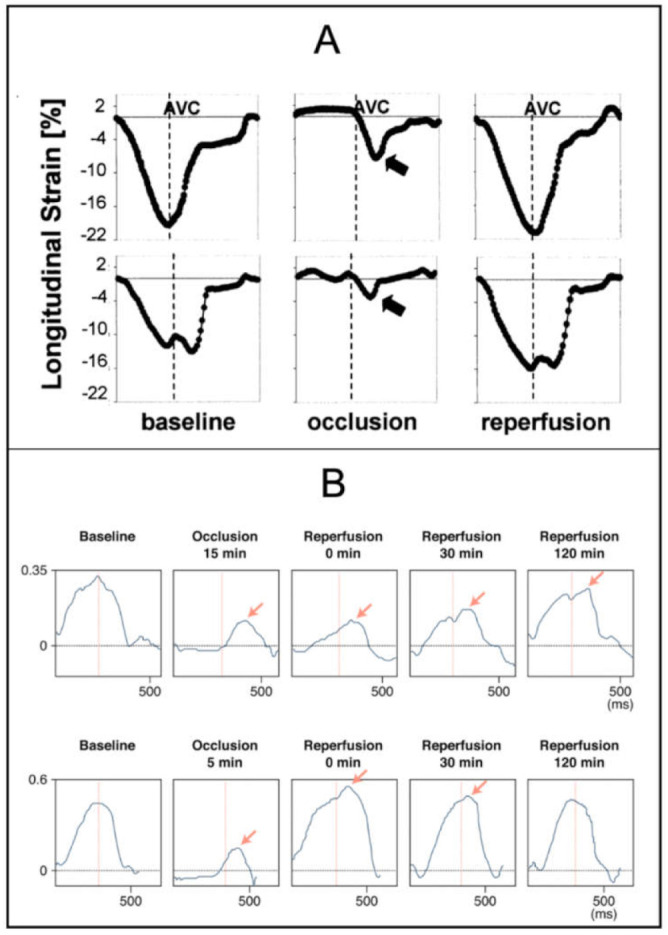
Deformation patterns during myocardial ischemia. Legend: (**A**) Longitudinal strain profiles in segment with normal wall motion (upper row) and segment with abnormal wall motion (lower row). Obtained before occlusion, 30 to 60 s after occlusion of LAD and 2 min after balloon reperfusion. The normal segment develops ESL and PSS after occlusion. The abnormal segment displays PSS at baseline, and the magnitude increases after occlusion. (**B**) Radial strain profiles of myocardial area exposed to 15 min (upper row) and 5 min (lower row) of coronary occlusion. Postsystolic thickening continues to appear after reperfusion in the segment exposed to 15 min of ischemia (indicating ‘ischemic memory’), whereas it disappears in the segment exposed to 5 min of ischemia. (**A**,**B**) are reprinted from Kukulski et al. [50] and Asanuma et al. [67] with permission from the American College of Cardiology (Elsevier). LAD: left anterior descending artery, AVC: aortic valve closure. PSS: postsystolic shortening, ESL: early systolic lengthening.

During acute ischemia, myocardial segments lengthen during early ejection phase (i.e., ESL), whereas unaffected segments continue to shorten. In general, this is accompanied by an overall decrease in systolic strain [46,47] and a concomitant increase in PSS [48,49,50] (Figure 3A). The magnitude of these changes is directly associated with the degree of stenosis and reduction in transmural blood flow [7,13,51,52,53,54,55,56,57]. During complete occlusion, overall deformation deteriorates; however, tethering to unaffected surrounding segments may still cause motion [32]. Upon reperfusion, both deformational patterns gradually diminish [55], although myocardial stunning can cause them to persist for a while [27]. In stunned segments with preserved elasticity, recovery of systolic deformation is paralleled by a concomitant decrease in PSS [58]. Thus, in a clinical setting, identification of PSS and ESL may be useful in patients with suspected ischemia [16,59,60], and it is proposed that up to 80% of patients with coronary artery disease show PSS [8]. A recent study from our group linked PSS to coronary artery calcium score [61], indicating that recognition of PSS potentially may aid in the selection of patients referred for cardiac computed tomography. As of today, an abundant number of studies support that PSS possibly may be superior to conventional echocardiographic parameters and systolic strain for differentiating ischemic versus non-ischemic segments [50,62,63,64,65]. Far fewer studies have assessed the usefulness of ESL in acute ischemia. As opposed to regional longitudinal strain, which has only gained limited clinical use due to questionable reproducibility, recognition of regional deformational patterns, including PSS and ESL [17,66], may be associated with localization of coronary occlusion and infarct site.

### 3.2. Predictor of Recovery

In terms of recovery, higher levels of PSS at baseline are associated with improved long-term systolic recovery [14,23,68,69]. Specifically, it was demonstrated that segments that showed PSS provoked during echocardiographic stress testing had a greater potential for recovery from revascularization [26,70,71]. For ESL, two studies found that the duration correlated with final infarct size and transmural extent of infarction as determined by magnetic resonance imaging [66,72]. Based on this, it is possible that deformational patterns contain useful information when assessed prior to revascularization. The clinical significance is that paradoxical deformation patterns possibly may guide the selection of patients who would benefit from revascularization procedures. Recovery is not only restricted to myocardial ischemia, as the presence of PSS is also associated with reverse remodeling and improved systolic function after long-term treatment with cardiac resynchronization therapy in heart failure patients [73,74].

### 3.3. Ischemic Memory Imaging

When exposed to transient ischemia (seconds to minutes) and later re-perfused, abnormal deformation continues for a period of time despite normalization of blood flow (Figure 3B). The delayed appearance of abnormal deformation may be useful in situations where acute chest pain has resolved prior to arrival in the hospital and is referred to as ischemic memory imaging [67,75,76]. Studies have demonstrated that PSS and ESL have greater accuracy compared with systolic strain for identifying ischemic memory [77,78]. Because pathological PSS decreases over time, whereas physiological PSS remains constant, serial assessment is necessary in patients with transient ischemia [6]. Although ischemic memory imaging was introduced nearly a decade ago, reference values of PSS and ESL in this field are undetermined, and the time interval for reliable detection after ischemia is unclear. 

### 3.4. Chronic Ischemia and Fibrosis

Chronic ischemia leads to myocyte loss and fibrosis, which affects tissue elasticity and deformation [27]. While subendocardial fibrosis reduces systolic deformation and induces PSS and ESL, segments with transmural fibrosis display no deformation [27]. This happens because the extent of deformation is directly related to the degree of fibrosis. Individuals with heart failure and reduced ejection fraction can therefore exhibit values of PSS within the range of healthy persons, as demonstrated by Ring et al. [45] (proposed natural history of PSS is depicted in Figure 1B).

### 3.5. Prognostic Value

More recently, our research group has shown the ability of PSS and ESL to predict adverse cardiovascular events in a spectrum of populations [17,37,38,69,79,80,81,82,83]. It was demonstrated that both deformational patterns yield prognostic information beyond that of conventional echocardiographic parameters and systolic strain, ranging from the general population [10,38] to persons with diabetes [82,83] and acute ischemia [17,69,79].

## 4. Future Directions

In the last two decades, the clinical utility of speckle tracking imaging has been studied extensively. Research has focused on standardization and solving inter-vendor differences, and today global longitudinal strain is one the most robust and reproducible strain parameters [84]. Considering this progress, new parameters are being studied, such as left atrial and right ventricular strain, and abnormal deformational patterns have attracted novel attention.

Even though assessment of PSS and ESL constitutes a niche within strain imaging, and their role in clinical practice still is undergoing development, they have been evaluated in a variety of study populations, mostly focusing on ischemia. Table 2 displays an overview of current research areas and clinical applicability. From a clinical perspective, being able to differentiate physiological from pathological PSS and ESL remains an important issue to resolve. Except for serial assessment, where relative changes are quantified, reference values in different populations need to be determined to advance and encourage clinicians to identify deformational patterns. This may pave the road for risk stratification models and for implementing assessment in clinical routine, in which echocardiography already constitutes a key examination. The introduction of three-dimensional echocardiography represents a new technology for strain imaging that has recently gained attention in clinical research. Precautions should be taken as to whether this imaging modality provides sufficient temporal and spatial resolution for accurately identifying such short-lived events as PSS and ESL [85].

Paradoxical deformation patterns are not solely restricted to the left ventricle. They also occur in the right ventricle, where deformation can extend beyond closure of the pulmonic valve. More specifically, this was observed in populations with increased right ventricular systolic pressure [86,87]. Given the more recent use of speckle tracking of the right ventricle [88], this represents a new localization for investigations of paradoxical deformation. Combined analysis of PSS and ESL have been proposed, including the lengthening–shortening ratio [30] and, more recently, the myocardial deformation index [78]. The rationale is that both patterns represent abnormality; yet, it remains to be determined if ESL always coexists in the presence of PSS and whether it is necessary to assess both patterns at the same time. PSS and ESL are influenced by loading conditions and should therefore be cautiously interpreted. A novel parameter, the myocardial work index (or global wasted work), which incorporates strain and blood pressure, may constitute a more solid approach to dealing with loading conditions, potentially making assessment of PSS and ESL by speckle tracking redundant. This should be investigated in future clinical studies.

At the present time a plethora of different echocardiographic indices exists, each reflecting different aspects of the cardiac cycle. Many of these are correlated and can cause unnecessary confusion. For clinical translation, there is a need to simplify the overabundance of parameters and develop risk models that take several measures into account at the same time. These risk models cannot replace conventional echocardiographic examinations, but they may help in assessing what the eye cannot see.

## 5. Conclusions

Analysis of myocardial strain has allowed detection of subtle abnormal deformation patterns, including PSS and ESL, that are difficult to detect visually and by conventional echocardiography. Assessment of these patterns may provide both diagnostic and prognostic information, ranging from the general population to acute and chronic ischemia and in patients undergoing cardiac resynchronization therapy. Even though research on this topic has recently expanded significantly, there are, as highlighted, several issues that still need to be resolved prior to implementing these patterns in clinical practice.

## Figures and Tables

**Table 1:** Criteria by Voigt et al. [8] to identify pathologic postsystolic shortening by strain derived from tissue Doppler imaging.

Criteria
1. Short-lived postsystolic shortening occurring during transient ischemia
2. Postsystolic shortening occurring when absolute ejection time peak strain > −7%
3. Postsystolic shortening occurring when −7% > ejection time peak strain > −18% and: - Exceeds 20% of maximum strain in cardiac cycle - Peak postsystolic shortening > 90 ms after aortic valve closure

**Table 2:** Research areas and potential clinical applicability of PSS and ESL.

Acute Ischemia	Transient Ischemia	Recovery	Prognosis
Tissue viability Degree of stenosis Coronary artery calcium score	Ischemic memory imaging	Identification of stunned myocardium Benefit of revascularization Cardiac resynchronization therapy	General population Diabetes Ischemia Heart failure

PSS: postsystolic shortening, ESL: early systolic lengthening.
